# Macular pigment optical density in a Brazilian sample

**DOI:** 10.1186/s40942-018-0107-1

**Published:** 2018-01-22

**Authors:** Letícia Pinto Coelho Jorge, Carlos Eduardo Gonçalves Pereira, Eduardo Jorge, Marcos Pereira de Ávila

**Affiliations:** 1 Instituto Panamericano da Visão, Street T 8 No. 171 Setor Marista, 74150-060 Goiânia, GO Brazil; 2 Universidade Federal de Goiás, Av 1 No. 355 Setor Universitário, 74605-020 Goiânia, GO Brazil

**Keywords:** Macular pigment, Macula lutea, Macular degeneration, Ethnic groups

## Abstract

**Background:**

To evaluate macular pigment optical density (MPOD) and to identify its determinants in a sample of Brazilian individuals.

**Methods:**

This was a cross-sectional study. One hundred three healthy individuals had both eyes photographed using a Visucam 500 digital fundus camera (Carl Zeiss Meditec, Jena, Germany) in combination with the MPOD module. Four variables were obtained: maximum MPOD, mean MPOD, MPOD volume, and MPOD area. Demographic data and information on lifestyle habits were also collected.

**Results:**

Mean MPOD was 0.14 density unit ± 0.05. MPOD was not influenced by gender, smoking history, or refractive error. MPOD was significantly higher among black individuals than among white and biracial individuals. There was a positive but low correlation between MPOD and age.

**Conclusion:**

This study found MPOD values to be similar to those found in European samples but lower than other studies performed on Asian and Australian samples. This is the first data regarding MPOD in a South American Population.

## Background

Macular pigment is composed of chemical substances known as xanthophylls, which include three isomers: lutein, zeaxanthin, and meso-zeaxanthin [1]. The macula lutea is the area where these yellow pigments are found [2, 3], particularly in the Henle fiber layer in the fovea centralis and the inner plexiform layer in the parafovea [4–6].

Macular pigments work as an optic filter for wavelengths below 550 nm and also have antioxidant properties. It is believed that they are a protective factor for diseases caused by oxidative stress, such as age-related macular degeneration [3].

The quantitative study of macular pigment and its distribution is possible through macular pigment optical density (MPOD). The methods for MPOD measurement can be divided into subjective and objective approaches. Subjective methods include heterochromatic flicker photometry (HFP) and motion photometry. Objective methods include reflectometry, autofluorescence, and Raman scattering [7].

MPOD can also be objectively evaluated using the Visucam 500 digital fundus camera (Carl Zeiss Meditec, Jena, Germany), which measures the reflectance of a blue light close to the macular pigment’s area of maximum absorption. This method produces a graph that shows the three-dimensional distribution of macular pigment and evaluates four variables: maximum MPOD, mean MPOD, MPOD volume, and MPOD area [8–10].

It is believed that different populations have different macular pigment distributions [11]. Other variables, such as age, sex, history of tobacco use, ethnicity, and refractive error also seem to influence MPOD values [12].

There is no literature on an average MPOD value for healthy individuals in a Brazilian sample. This information is essential, since it will serve as a point of reference for future studies of macular pigments and macular degeneration in this population.

The purpose of this study was to determine the mean MPOD value in a sample of the Brazilian population and to evaluate the influence of sex, age, ethnicity, smoking history and refractive status on MPOD values in this sample.

## Methods

This study was approved by the Ethics Committee of the School of Medicine of the Federal University of Goiás and obeys the Declaration of Helsinki.

A cross-sectional study was performed. One hundred fifty healthy subjects between the ages of 18 and 76 were selected from the Panamerican Institute for Eyesight (Instituto Panamericano da Visão), in the city of Goiânia, Goiás State, Brazil. All subjects were informed of the study and signed an informed consent form.

All subjects received a complete ophthalmologic exam, including visual acuity testing, testing to determine refractive error, biomicroscopy, fundoscopy, and tonometry. Subjects with a positive spherical equivalent above 0.25 D were considered hyperopic; those with a negative spherical equivalent were considered myopic, and those with a spherical equivalent between 0.00 and + 0.25 D were classified as emmetropic. Demographic data was collected on sex, age, and ethnicity, the latter of which was self-reported according to the classification used by the Brazilian Institute of Geography and Statistics (IBGE), which offers the categories of white, black, biracial, and indigenous [13]. Data on lifestyle was also collected, including individuals’ tobacco use (smokers were defined as individuals who had smoked at least one cigarette per day for the 6 months prior) [14], and information on use of vitamin supplements. The inclusion criteria were best corrected visual acuity above 84 letters according to the Early Treatment Diabetic Retinopathy Study (ETDRS) [15] chart, clear ocular media and spherical equivalent between ± 4.0 D. The exclusion criteria were the presence of any ocular disease or opaque ocular media (clinically significant cataract or cataract leading to a decrease in visual acuity), intraocular surgery or previous trauma, use of vitamin supplements, diabetes, hypertension, or other metabolic diseases. After these criteria were applied, a total of 103 participants were admitted to the study.

All subjects had both eyes dilated using tropicamide 0.5 mg/mL and, after 30 min, both eyes were photographed using the Visucam 500 system (Carl Zeiss Meditec, Jena, Germany) combined with the MPOD module. MPOD was calculated at 7 degrees of eccentricity, at which point the highest concentration of xanthophyll was reached. All images were collected by the same technician, under the same light conditions, with the same flash intensity and after mydriasis. The MPOD analysis provided information on maximum MPOD, mean MPOD, MPOD volume, and MPOD area, as well as a colored map and a three-dimensional pigment distribution profile. Maximum and mean MPOD were measured in density units (d.u.). The value of MPOD volume corresponds to the sum of the optical density values at all points and is given in d.u. degrees^2^. The value of MPOD area corresponds to the area where pigment is detected and is given in degrees^2^ [10].

The software Statistical Package for Social Science (SPSS) for Windows (version 21.0) was used for data analysis.

The category variables are provided in a table with absolute values (f) and percentages (%) The continuous variables are presented as a mean ± SD and with a confidence interval of 95%.

Wilcoxon test for paired data was used to check for the presence of a significant difference between the MPOD variables (volume, area, maximum, and mean) measured in the right and left eye.

Mann–Whitney test for independent data was used to check for the existence of a significant difference between the four MPOD variables (volume, area, maximum, and mean) when correlated with sex, ethnicity, and tobacco use. Mann–Whitney test was also employed to determine whether there was a significant difference in the MPOD variables (volume, area, maximum, and mean) when correlated with refractive error (emmetropia, myopia and hyperopia).

Linear regression analysis was used to determine whether there were any correlations between the MPOD variables and age.

All tests employed a 95% confidence interval and defined significance as *p* < 0.05.

For sample calculations, Table 1 of page 263 of the study entitled “Macular Pigment Optical Density in a Central European Population” was used [12]. The test used a standard deviation of 0.7%, a level of significance of 5%, and a test power of 80%, with a minimum sample of 79 eyes.

## Results

Two hundred six eyes of 103 subjects were analyzed.

The distribution of the subjects according to sex, ethnicity, and history of tobacco use is shown in Table 1.

**Table 1:** Distribution of subjects by sex, ethnicity, and history of tobacco use

Variable	Frequency (n = 103)	%
*Sex*		
Female	66	64.1
Male	37	35.9
*Ethnicity*		
White	30	29.1
Black	25	24.3
Biracial	48	46.6
*Tobacco use*		
Yes	22	21.3
No	81	78.7

The mean value of the four MPOD variables (volume, area, maximum, and mean) are shown in Table 2.

**Table 2:** Mean and standard deviation of macular pigment optical density: volume, area, maximum and mean

Variable	Mean ± SD	95% CI
MPOD volume	8837.74 ± 2674.98	8470.29–9205.20
MPOD area	62,269.03 ± 11,724.23	60,658.50–63,879.57
Max MPOD	0.39 ± 0.07	0.38–0.40
Mean MPOD	0.14 ± 0.05	0.14–0.15

Values of maximum and mean MPOD in density units (d.u.); volume measured in d.u. degrees^2^; area measured in degrees^2^

MPOD, macular pigment optical density; Max, maximum; CI, confidence interval

The mean values for the MPOD variables (volume, area, maximum, and mean) were correlated with ethnicity, and the comparisons are found in Tables 3 and 4. All MPOD variables (volume, area, maximum and mean) were significantly higher among black individuals than among biracial and white subjects. There were no statistically significant differences between MPOD variables among white and biracial individuals.

**Table 3:** Mean MPOD values (volume, area, maximum and mean) and their correlations with ethnicity

Variable	White	Black	Biracial
	Mean ± SD	Mean ± SD	Mean ± SD
MPOD volume	8425.57 ± 2028.56	10,884.92 ± 2266.50	8029.12 ± 2030.03
MPOD area	61,723.95 ± 13,150.87	68,496.14 ± 10,031.52	59,366.43 ± 10,401.73
Max MPOD	0.39 ± 0.08	0.43 ± 0.05	0.37 ± 0.07
Mean MPOD	0.13 ± 0.03	0.16 ± 0.02	0.14 ± 0.06

Values of maximum and mean MPOD measured in density units (d.u.); volume measured in d.u. degrees^2^; area measured in degrees^2^

MPOD, macular pigment optical density; Max, maximum; SD, standard deviation

**Table 4:** Macular pigment optical density variables (volume, area, maximum and mean) and their correlations with ethnicity

Variable	White × black	White × biracial	Black × biracial
	*p*	*p*	*p*
MPOD volume	< 0.001*	0.907	< 0.001*
MPOD area	0.003*	0.331	< 0.001*
Max MPOD	< 0.001*	0.850	< 0.001*
Mean MPOD	< 0.001*	0.359	< 0.001*

Mann–Whitney test for independent data with a 95% confidence interval

MPOD, macular pigment optical density; max, Maximum

*Level of significance of *p* < 0.05

**Fig. 1:**
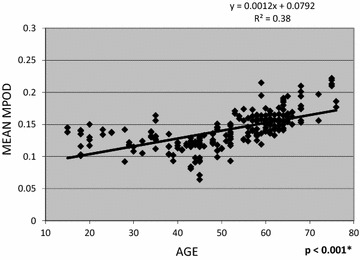
Linear regression of age and mean macular pigment optical density. 95% confidence interval; level of significance of p < 0.05

**Fig. 2:**
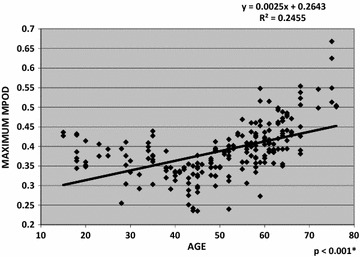
Linear regression of age and maximum macular pigment optical density. 95% confidence interval; level of significance of p < 0.05

The linear regression of age and mean MPOD is shown in Fig. 1. There was a low but positive correlation between mean MPOD and age (R^2^ = 0.37996, *p* < 0.001). The linear regression of age and maximum MPOD is shown in Fig. 2. There was a low but positive correlation between maximum MPOD and age (R^2^ = 0.2455, *p* < 0.001). There was no correlation between the other MPOD variables and age.

There was no statistically significant difference in the comparisons between the four MPOD variables (volume, area, maximum, and mean) and sex. There was also no statistically significant difference between smokers and non-smokers.

There were no statistically significant differences in MPOD values when the results for myopic, emmetropic, and hyperopic subjects were compared. There was also no statistically significant difference between the MPOD values obtained when the results for left eyes and right eyes were compared.

## Discussion

It is believed that different populations have different distributions of macular pigments [11]. Studies to determine MPOD in certain populations have been performed in many locations, including China [14], Australia [16], Central Europe [12], and South Asia [17]. Mean MPOD values have varied substantially from one study to the other. Mean MPOD obtained from a sample from Central Europe (0.126 d.u. ± 0.004) is very close to this Brazilian sample (0.14 d.u. ± 0.05). The remaining studies found higher values: mean MPOD in Australia was 0.41 d.u. ± 0.20; mean MPOD in South Asia was 0.43 d.u. ± 0.14; and mean MPOD in China was 0.303 d.u. ± 0.097. A possibility for this difference could be the technique used to determine MPOD. This study and the one performed in Central Europe were the only ones to use the Visucam 500 (Carl Zeiss Meditec, Jena, Germany). The studies performed in South Asia and Australia used the subjective HFP method. However, the literature shows a strong correlation between the values obtained through the reflectometry technique and the HFP [18, 19]. Var der Veen et al. [18] propose a corrective factor, with the addition of 0.05 d.u. to the value obtained through HFP when it is compared to reflectometry values. In this case, the difference between the values obtained in this study and the other researches using the HFP technique would be even higher. This finding indicates that there are other factors leading to the differences found between populations. Another piece of evidence that confirms this hypothesis is the fact that the Chinese population study used the reflectometry technique and a Visucam 200 (Carl Zeiss Meditec, Jena, Germany) to measure MPOD and found higher values than those found in this study. Of course, a population is not only defined by its geographical location, but also by lifestyle and diet habits, as well as by racial and genetic miscegenation. Those factors could have influenced the different mean MPOD values between different populations.

This study did not find significant differences in the MPOD values between men and women. In fact, most studies found no correlations between MPOD values and sexes [12, 14, 16, 20], although higher MPOD values have previously been found both in men [17] and women [21].

In this study, all MPOD variables (volume, area, maximum and mean) were found to be significantly higher in black subjects than in biracial and white individuals. Studies show that white individuals have lower central MPOD values than non-whites, including Southern Asian [17, 22, 23] samples and a sample of black subjects [11]. Those studies corroborate our findings.

History of tobacco consumption does not seem to influence MPOD values [12, 14, 16, 17]. The results found herein were consistent with those of other studies. A small number of studies have found lower values among the smokers [24, 25].

Few studies have evaluated the influence of refractive error on MPOD and have not found a significant difference between myopic, emmetropic, and hyperopic individuals [26]. This study found the same results. Tong et al. [27] observed a negative correlation between MPOD and axial length in myopic subjects. However, this correlation was found only in the group with lengths greater than 26 mm, cases which were classified as high myopia. The current study did not include spherical equivalents above 4.0 D and, although axial length was not measured herein, it is likely that this study would not have included subjects with extreme axial lengths. That information could justify the lack of significant differences between myopic, emmetropic and hyperopic individuals.

No significant differences were found in MPOD values obtained from right eyes versus left eyes. This is in line with findings described in the literature [28–30]. In fact, Davey et al. [31] report that MPOD values found in one eye can work as a predictive factor of the values in the other eye with 89% precision and that, clinically, the measurements of only one eye could be an indicator of MPOD measurements in healthy individuals.

This study found a low but positive correlation between mean and maximum MPOD and age. Other MPOD variables (area and volume) were not correlated with age. There is no consensus in literature regarding the influence of age on MPOD values. Berendschot et al. [32] found an increase in MPOD with age in individuals over 55 years of age. Ciulla et al. [33] did not find a correlation between age and MPOD in a group of individuals aged 18–50. Lima [34] observed that MPOD values peaked between 45 and 50 years of age, followed by a gradual decrease after the age of 60. Ji et al. [14] reported a decrease in MPOD as age increased. It is important to note that the opacity in media caused by age-related cataracts could lead to lower MPOD values when MPOD is measured using reflectometry, and that the presence of intraocular lens implants could also influence the measurements [10]. This study did not include pseudophakic subjects or individuals with clinically significant cataracts. However, the study did include older subjects whose senile lenses could have influenced MPOD measurements. Even so, a positive correlation between MPOD and age was found. A possible explanation for this finding is that lipofuscin found in the retinal pigment epithelium presents a pattern of light absorption similar to the absorption spectrum of the macular pigment. This substance could also interfere in MPOD measurements obtained using reflectometry and autofluorescence [20, 25]. Lupofuscin increases with age, a fact which could explain the increase in MPOD associated with aging.

This study presents some limitations. The subjects’ diets were not evaluated, and the amount of serum xanthophylls was not measured. These factors could affect MPOD [2, 12]. Despite the limitations, this study provides new data on MPOD in a Brazilian sample, as well as the determining factors for the results.

## Conclusions

Mean MPOD was 0.14 density unit ± 0.05. MPOD was not influenced by gender, smoking history, or refractive error. MPOD was significantly higher among black individuals than among white and biracial individuals. There was a positive but low correlation between mean and maximum MPOD and age.

This is the first data regarding MPOD in a South American Population and establishes reference values for future studies.

## Abbreviations

MPOD: macular pigment optical density
HFP: heterochromatic flicker photometry
IBGE: Brazilian Institute of Geography and Statistics
ETDRS: Early Treatment Diabetic Retinopathy Study
D.U.: density unit
SPSS: statistical package for social science

## References

[1] Ahmed SS, Lott MN, Marcus DM (2005). The macular xanthophylls. Surv Ophthalmol.

[2] Bone RA, Landrum JT, Gerra LH (2003). Lutein and zeaxanthin dietary supplements raise macular pigment density and serum concentrations of these carotenoids in humans. J Nutr.

[3] Beatty S, Boulton M, Henson D (1999). Macular pigment and age related macular degeneration. Br J Ophthalmol.

[4] Bone RA, Landrum JT (1992). Distribution of macular pigments components, zeaxanthin and lutein, in human retina. Methods Enzymol.

[5] Rapp LM, Maple SS, Choi JH (2000). Lutein and zeaxanthin concentrations in rod outer segment membranes from perifoveal and peripheral human retina. Invest Ophthalmol Vis Sci.

[6] Trieschmann M, van Kuijk FJ, Alexander R (2008). Macular pigment in the human retina: histological evaluation of localization and distribution. Eye.

[7] Howells O, Eperjesi F, Bartlett H (2011). Measuring macular pigment optical density in vivo: a review of techniques. Graefes Arch Clin Exp Ophthalmol.

[8] Ivins P, McArthur C. Technology on test. A report on an objective method of measuring macular pigment using the new Zeiss Visucam 200. Optician. 2011;18–22.

[9] Visucam digital fundus camera: Addendum zum Dokumentensatz. Hinweise zur Benutzung des optionalen MPD-Moduls. Jena: Carl Zeiss Meditec AG; 2010.

[10] Schweitzer D, Jentsch S, Dawczynski J (2010). Simple and objective method for routine detection of the macular pigment xanthophyll. J Biomed Opt.

[11] Wolf-Schnurrbusch UE, Roosli N, Weyermann E (2007). Ethnic differences in macular pigment density and distribution. Invest Ophthalmol Vis Sci.

[12] Pipis A, Touliou E, Augustin AJ (2013). Macular pigment optical density in a Central European population. OSLI.

[13] Instituto Brasileiro de Geografia e Estatística (IBGE): Características Étnico-raciais da População: Classificações e identidades. Rio de Janeiro, Brazil; 2013.

[14] Ji Y, Zhang X, Wu K (2015). Macular pigment optical density in a healthy Chinese population. Acta Ophthalmol.

[15] Falkenstein I, Cochran D, Azen S (2008). Comparison of visual acuity in macular degeneration patients measured with Snellen and early treatment diabetic retinopathy study charts. Ophthalmology.

[16] Abell RG, Hewitt AW, Andric M (2014). The use of heterochromatic flicker photometry to determine macular pigment optical density in a healthy Australian population. Graefes Arch Clin Exp Ophthalmol.

[17] Howells O, Eperjesi F, Bartlett H (2013). Macular pigment optical density in young adults of South Asian origin. Invest Ophthalmol Vis Sci.

[18] Van Der Veen RL, Berendschot TT, Makridaki M (2009). Correspondence between retinal reflectometry and a flicker-based technique in the measurement of macular pigment spatial profiles. J Biomed Opt.

[19] Van Der Veen RL, Berendschot TT, Hendrikse F (2009). A new desktop instrument for measuring macular pigment optical density based on a novel technique for setting flicker thresholds. Ophthalmic Physiol Opt.

[20] Dietzel M, Zeimer M, Heimes B (2011). Determinants of macular pigment optical density and its relation to age-related maculopathy: results from the Muenster Aging and Retina Study (MARS). Invest Ophthalmol Vis Sci.

[21] Nolan JM, Kenny R, O’regan C (2010). Macular pigment optical density in an ageing Irish population: the Irish longitudinal study on ageing. Ophthalmic Res.

[22] Huntjens B, Asaria TS, Dhanani S (2014). Macular pigment spatial profiles in South Asian and white subjects. Invest Ophthalmol Vis Sci.

[23] Raman R, Rajan R, Biswas S (2011). Macular pigment optical density in a South Indian population. Invest Ophthalmol Vis Sci.

[24] Hammond BR, Caruso-Avery M (2000). Macular pigment optical density in a Southwestern sample. Invest Ophthalmol Vis Sci.

[25] Delori FC, Goger DG, Hammond BR (2001). Macular pigment density measured by autofluorescence spectrometry: comparison with reflectometry and heterochromatic flicker photometry. J Opt Soc Am A Opt Image Sci Vis.

[26] Zheng W, Zhang Z, Jiang K (2013). Macular pigment optical density and its relationship with refractive status and foveal thickness in Chinese school-aged children. Curr Eye Res.

[27] Tong N, Zhang W, Zhang Z (2013). Inverse relationship between macular pigment optical density and axial lenght in Chinese subjects with myopia. Graefes Arch Clinic Exp Ophthalmol.

[28] Beatty S, Murray IJ, Henson DB (2001). Macular pigment and risk for age-related macular degeneration in subjects from a Northern European population. Invest Ophthalmol Vis Sci.

[29] Iannaccone A, Mura M, Gallaher KT (2007). Macular pigment optical density in the elderly: findings in a large biracial Midsouth population sample. Invest Ophthalmol Vis Sci.

[30] Snodderly DM, Mares JA, Wooten BR (2004). CAREDS Macular Pigment Study Group Macular pigment measurement by heterochromatic flicker photometry in older subjects: the carotenoids and age-related eye disease study. Invest Ophthalmol Vis Sci.

[31] Davey PG, Alvarez SD, Lee JY (2016). Macular pigment optical density: repeatability, intereye correlation, and effect of ocular dominance. Clin Ophthalmol.

[32] Berendschot TT, Willemse-Assink JJ, Bastiaanse M (2002). Macular pigment and melanin in age-related maculopathy in a general population. Invest Ophthalmol Vis Sci.

[33] Ciulla TA, Curran-Celantano J, Cooper DA (2001). Macular pigment optical density in a midwestern sample. Ophthalmology.

[34] Lima VC, Rosen RB, Prata TS (2013). Association of age and macular pigment optical density using dual-wavelength autofluorescence imaging. Clin Ophthalmol.

